# Inhibition of Cytochrome P450 Activities by* Sophora flavescens* Extract and Its Prenylated Flavonoids in Human Liver Microsomes

**DOI:** 10.1155/2019/2673769

**Published:** 2019-03-13

**Authors:** Daeun Yim, Min Jung Kim, Yumi Shin, Su-Jun Lee, Jae Gook Shin, Dong Hyun Kim

**Affiliations:** Department of Pharmacology and Pharmacogenomics Research Center, Inje University College of Medicine, Busan 614-735, Republic of Korea

## Abstract

*Sophora flavescens *possesses several pharmacological properties and has been widely used for the treatment of diarrhea, inflammation, abscess, dysentery, and fever in East Asian countries.* S. flavescens* is a major source of prenylated flavonoids, such as sophoraflavone and kushenol. In this study, we examined the effects of* S. flavescens* extract and its prenylated flavonoids on cytochrome P450 (CYP) isoform activity in human liver microsomes. The extract inhibited CYP2C8, CYP2C9, CYP2C19, and CYP3A activities, with IC_50_ values of 1.42, 13.6, 19.1, and 50 *µ*g/mL, respectively. CYP2B6 was only inhibited in human liver microsomes preincubated with the extract. CYP3A4 was more strongly inhibited by the extract in the presence of NADPH, suggesting that the extract may inhibit CYP2B6 and CYP3A4 via mechanism-based inactivation. Prenylated flavonoids also inhibited CYP isoforms with different selectivity and modes of action. Kushenol I, leachianone A, and sophoraflavone G inhibited CYP2B6, whereas kushenol C, kushenol I, kushenol M, leachianone A, and sophoraflavone G inhibited CYP3A4 via mechanism-based inhibition. Our results suggest that* S. flavescens *may contribute to herb–drug interactions when coadministered with drugs metabolized by CYP2B6, CYP2C8, CYP2C9, and CYP3A4.

## 1. Introduction

The dried roots of* Sophora flavescens*, a traditional Chinese medicine, have been widely used in Korea, Japan, and China for the treatment of solid tumors and inflammatory disease [1]. Moreover,* S. flavescens* exerts diverse pharmacological properties including antianaphylaxis, antimicrobial, immunoregulatory, and cardioprotective activities [2]. These therapeutic effects of* S. flavescens* may be derived from complex interactions among its various constituents. Phytochemical analysis revealed the presence of quinolizidine alkaloids and prenylated flavonoids in* S. flavescens *[3, 4]. These two chemicals have been shown to exert a wide spectrum of pharmacological effects, such as anti-inflammatory, antitumor, antimalarial, and antiviral effects [2, 5–8].

Recently, herb–drug interactions have drawn considerable attention, as they can lead to serious adverse effects or diminished drug efficacy. Herb–drug interactions may occur via modulation of hepatic and intestinal cytochrome P450 (CYP) drug-metabolizing enzymes and drug transporters [9, 10]. CYPs play a central role in the metabolism and elimination of xenobiotics including drugs, environmental pollutants, and food ingredients. Previous studies have reported potential herb–drug interactions for St. John's wort [11], gingko biloba [12], goldenseal [13], and ginseng [14] via induction and/or inhibition of CYPs. Furthermore, several* in vitro* and* in vivo* studies have shown that* S. flavescens* extract modulates CYP activities, both induction and inhibition depending on the experimental design. In rat models, oral administration of* S. flavescens* extract resulted in induction of CYP2D and inhibition of CYP1A2 and CYP2C [15]. Sophocarpine from* S. flavescens *was reported to inhibit CYP3A4 and CYP2C9 in human liver microsomes [16]. Induction of CYP1A, CYP2B1/2, CYP2C11, and CYP3A following treatment with* S. flavescens* was observed in rats and mice, and the alkaloids matrine and oxymatrine contributed to induction of CYP isoforms [17–19]. In rats, treatment with* S. flavescens *extract significantly reduced the exposure of indinavir, a substrate of CYP3A and P-glycoprotein [20]. Nevertheless, studies evaluating the* in vitro* effects of* S. flavescens* extract and/or its flavonoids on human CYP isoform activity are limited.

The aim of the present study was to evaluate the effects of* S. flavescens* extract and its prenylated flavonoids on the activity of eight CYP isoforms in human liver microsomes, to further our understanding of the potential effects of* S. flavescens* on drugs metabolized primarily by CYP enzymes. We demonstrated that* S. flavescens* extract reversibly inhibited the activities of CYP2C8, CYP2C9, and CYP2C19 whereas it inhibited CYP2B6 and CYP3A4 in a mechanism-based inactivation manner.

## 2. Materials and Methods

### 2.1. Materials

*S. flavescens* root extract was purchased from the Korea Plant Extract Bank (Chungbuk, Korea). The extract was prepared by extraction with a 70% ethanol solution. Glucose 6-phosphate, *β*-NADP+, glucose-6-phosphate dehydrogenase, phenacetin, coumarin, bupropion, diclofenac,* S*-mephenytoin, dextromethorphan, and chlorpropamide were purchased from Sigma Aldrich (St. Louis, MO, USA); rosiglitazone and midazolam from Toronto Research Chemicals (Toronto, ON, Canada); and kushenols A, C, I, and M, leachianone A, and sophoraflavone G from Core Sciences (Seoul, Korea). Pooled human liver microsomes, and baculovirus-insect cell expressed 2B6 and 3A4 were purchased from BD Gentest (Woburn, MA, USA). All other reagents were of the highest grade commercially available.

### 2.2. CYP Inhibition Assay

The incubation mixture consisted of 0.5 mg/mL human liver microsomes or 20 pmol/mL recombinant CYPs,* S. flavescens* extract (0.1–100 *μ*g/mL), and/or prenylated flavonoids (1–100 *µ*M), probe substrates for each CYP isoform, and an NADPH-generating system (3.3 mM glucose-6-phosphate, 1.3 mM *β*-NADP^+^, 3.3 mM MgCl_2_, and 1.0 U/mL glucose-6-phosphate dehydrogenase) in a total volume of 200 *μ*L potassium phosphate buffer (0.1 M, pH 7.4). As shown in Table 1, the probe substrates used in this experiment were 50 *μ*M phenacetin for CYP1A2, 5 *μ*M coumarin for CYP2A6, 50 *μ*M bupropion for CYP2B6, 10 *μ*M paclitaxel for CYP2C8, 100 *μ*M tolbutamide for CYP2C9, 10 *μ*M* S*-mephenytoin for CYP2C18, 5 *µ*M dextromethorphan for CYP2D6, 50 *µ*M chlorzoxazone for CYP2E1, and 5 *µ*M midazolam for CYP3A4 as described previously [21]. The reaction was initiated by the addition of an NADPH-generating system, followed by incubation in a water bath at 37°C for 20 minutes. The reaction was stopped by the addition of 200 *μ*L acetonitrile and 100 *μ*M chlorpropamide. The samples were then centrifuged at 13,000* g* for 5 minutes. The supernatants from each reaction were analyzed by LC-MS/MS. To determine if the extract or prenylated flavonoids were irreversible inhibitors of the various CYP isoforms, human liver microsomes were preincubated with the extract or prenylated flavonoids in the presence of an NADPH-generating system at 37°C for 30 minutes. The reaction was initiated by the addition of a CYP probe substrate, followed by a 20-minute incubation; the reaction was stopped by the addition of a 200 *μ*L internal standard solution (100 *μ*M chlorpropamide in acetonitrile). The samples were then centrifuged at 13,000* g* for 5 minutes. The supernatants from each reaction were analyzed by LC-MS/MS.

### 2.3. Inactivation Assay

To characterize the time- and concentration-dependent inhibition of CYP3A4 by* S. flavescens *extract and kushenol I, an inactivation study was performed using human liver microsomes and recombinant CYP3A4. Human liver microsomes (1 mg/mL) or recombinant CYP3A4 (100 pmol/mL) was incubated with various concentrations of* S. flavescens* extract and kushenol I. The reaction mixture was incubated at 37°C for 5 minutes prior to initiation of the reaction by addition of an NADPH-generating system. Following 0, 5, 10, 20, or 30 minutes of incubation, a 10 *μ*L aliquot from each reaction mixture was added to a second reaction containing 10 *μ*M midazolam, an NADPH-generating system, and 0.2 M potassium phosphate buffer (pH 7.4), at a total reaction volume of 100 *μ*L. After 20 minutes, the 1-hydroxymidazolam formed in the reaction was analyzed by LC-MS/MS.

### 2.4. HPLC-MS/MS Analysis

Chromatography was performed using the Agilent 1100 Series LC system (Agilent, Santa Clara, CA, USA), which consisted of an autosampler, binary pump, and column oven. The HPLC system was coupled to the 4000 QTRAP triple-quadrupole mass spectrometer (AB Sciex, Foster City, CA, USA) equipped with an electrospray ionization source. The turbo ion-spray interface was operated in positive-ion mode using nitrogen as the nebulizing agent, turbo spray, and curtain gas, which were set to optimum values of 40, 50, and 20 psi, respectively. The turbo gas temperature was set to 600°C, and the electrospray ionization needle voltage was programmed to 5,500 V. Quadrupoles Q1 and Q3 were set to unit resolution.

For the quantitation of prenylated flavonoids, each flavonoid was dissolved in methanol to prepare the standard stock solution (1 mg/mL). Calibration and quality control samples were prepared by serial dilution of stock solutions to known concentration.* S. flavescens* extract was also dissolved in methanol. An aliquot of the sample (100 *μ*L) was then spiked with 5 *μ*L of a digoxin solution (internal standard, 100 *μ*g/mL) and filtered through a 0.22 *μ*m membrane filter. A 5 *μ*L aliquot of each sample was injected into the LC-MS/MS. Separation was done using the Luna C18 column (100 × 2.0 mm, 3.0 *μ*m, Phenomenex, Torrance, CA, USA). The mobile phase consisted of (A) 0.1% formic acid and (B) 100% acetonitrile containing 0.1% formic acid. Stepwise liner gradient elution was performed as follows: 30% B at 0 minutes, 60% B at 10 minutes, 80% B at 15 minutes, maintenance for 5 minutes, and return to 30% B at 21 minutes. The flow rate was 0.2 mL/min. Detection of the ions was performed by monitoring the transition of m/z 409.2 → 164.9 for kushenol A, 439.2 → 365.0 for kushenol C, 455.2 → 178.9 for kushenol I, 509.2 → 301.0 for kushenol M, 439.2 → 164.9 for leachianone A, 425.2 → 165.0 for sophoraflavone G, and 798 → 97 for digoxin. Calibration curves were linear (*r*^2^ > 0.997) over the concentration range between 10 and 500 ng/mL. The lower limit of quantitation was set to 10 ng/mL for all prenylated flavonoids. The relative standard deviations for intra- and interday precision over the concentration range for the flavonoids were lower than 12.0% with accuracies between 86.0 and 108.6%.

The analysis of the primary metabolites produced by the CYP isoforms from selective substrates was done by the validated method described elsewhere [22] with minor modification. Separation was performed using a Luna C18 column (30 × 2.0 mm, 3 *μ*m, Phenomenex). The mobile phase consisted of (A) 0.1% formic acid and (B) 100% acetonitrile containing 0.1% formic acid. A liner gradient elution from a 15% to 80% solvent (B) was performed for 2.6 minutes following reequilibration for 5 minutes at a flow rate of 0.2 mL/min. Analytes were quantified by multiple-reaction monitoring with specific precursor/product ion transitions. Detection of ion values was performed by monitoring the transitions of m/z 152 → 110 for acetaminophen, 163 → 107 for 7-OH-coumarin, 256 → 238 for 6-OH-bupropion, 374 → 151 for OH-rosiglitazone, 312 → 230 for 4-OH-mephenytoin, 258 → 157 for dextrorphan, 342 → 175 for 1-OH-midazolam, and 277 → 175 for chlorpropamide. Chlorpropamide was used as an internal standard. Data acquisition and processing were performed using the Analyst software (ver. 1.4.1; Applied Biosystems, Foster City, CA, USA).

### 2.5. Data Analysis

CYP isoform activity in the presence of* S. flavescens* extract and prenylated flavonoids was expressed as a percentage of the corresponding value in the control. The IC_50_ values were calculated by nonlinear least square regression analysis using WinNonlin, ver. 2.1 (Pharsight, Mountain View, CA, USA). The* K*_*i*_ and* k*_*inact*_ values were calculated using a secondary double reciprocal plot. The natural logarithm of remaining enzyme activity is plotted against the preincubation time (Figure 5). The observed inactivation rate constants (*k*_*obs*_) were obtained by the slopes of the initial log-linear phases.* K*_*i*_ and* k*_*inact *_were calculated using the following equation: 1/*k*_*obs*_ = 1/*k*_*inact*_ + K_i_/*k*_*inact*_ · 1/[I], where [I] denotes concentration of inhibitor.

## 3. Results

### 3.1. Relative Levels of Six Prenylated Flavonoids in* S. flavescens* Extract

**Figure 1:**
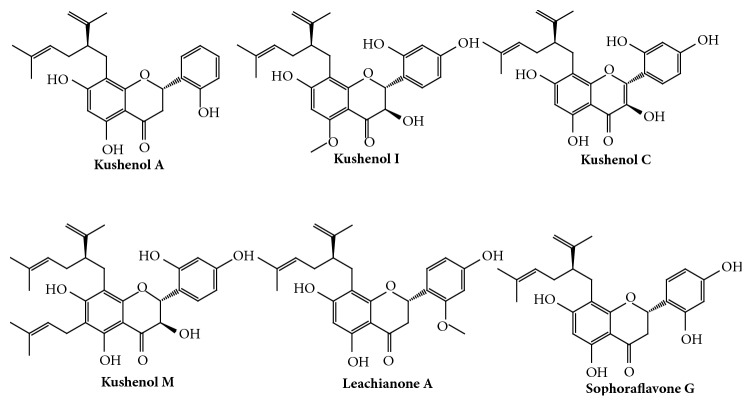
Structure of prenylated flavonoids in* S. flavescens *extract.

**Figure 2:**
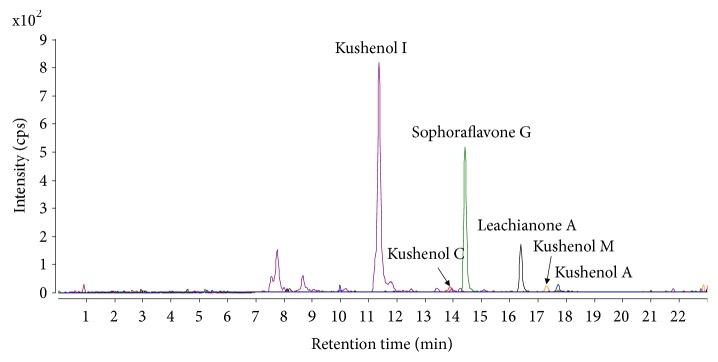
LC-MS/MS selected ion chromatogram of prenylated flavonoids in* S. flavescens *extract.

Various flavonoids have been detected in* S. flavescens* [2]. Because prenylated flavonoids act as inhibitors of CYP isoforms [23], the CYP inhibitory potential of six prenylated flavonoids (kushenol A, kushenol C, kushenol I, kushenol M, leachianone A, and sophoraflavone G) from* S. flavescens* was evaluated in human liver microsomes (Figure 1). The relative levels of flavonoids in* S. flavescens *extract were determined using LC-MS/MS. A reconstituted MRM chromatogram obtained from the* S. flavescens *extract is presented in Figure 2. All six prenylated flavonoids were detected in the extract. The relative levels of kushenol A, kushenol C, kushenol M, kushenol I, leachianone A, and sophoraflavone G were 0.08%, 0.02%, 0.10%, 1.17%, 0.51%, and 1.07%, respectively. Kushenol I and sophoraflavone G were the most abundant prenylated flavonoids detected in the extract.

### 3.2. Inhibition of CYP Isoforms by* S. flavescens* Extract and Its Prenylated Flavonoids

**Figure 3:**
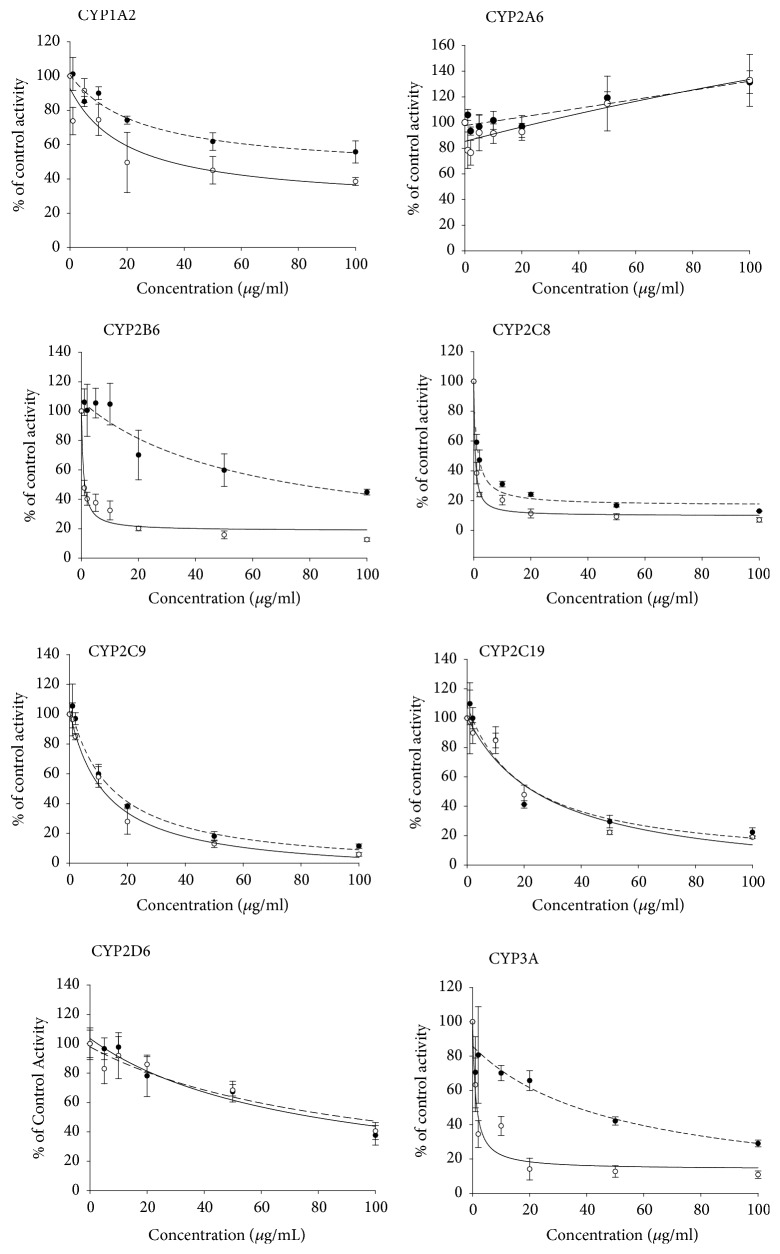
Concentration-dependent inhibition of CYP isoforms by* S. flavescens* extract and its prenylated flavonoids in human liver microsomes with (○) or without (●) preincubation in the presence of an NADPH-generating system. CYP activities are expressed as the relative percentage of the activity in the control. Each data point represents the mean of triplicate experiments.

**Figure 4:**
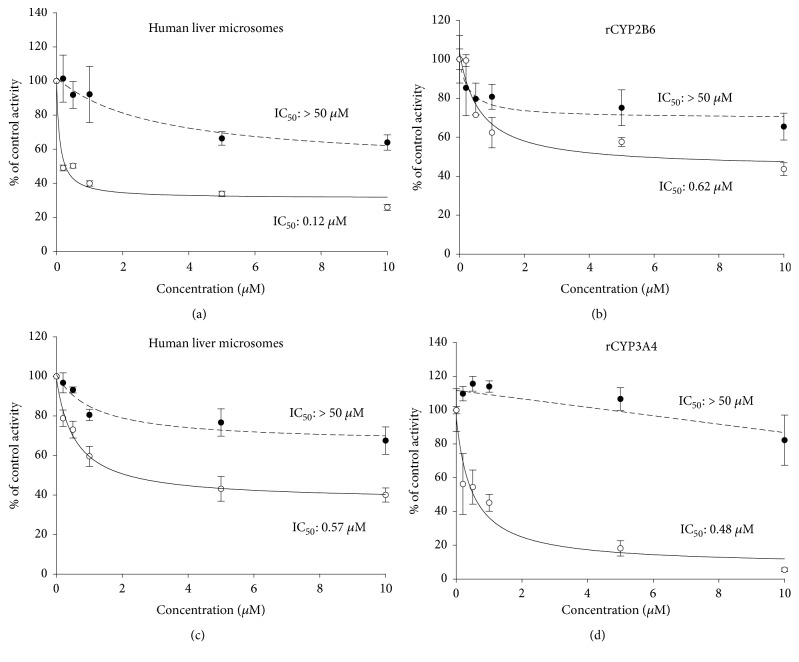
Concentration-dependent inhibition of bupropion 6-hydroxylation and midazolam 1'-hydroxylation by kushenol I with (○) or without (●) preincubation in the presence of an NADPH-generating system; microsomal- (a) and recombinant CYP2B6- (b) mediated bupropion 6-hydroxylation and microsomal- (c) and recombinant CYP3A4- (d) mediated midazolam 1'-hydroxylation. CYP activities are expressed as the relative percentage of the activity in the control. Each data point represents the mean of triplicate experiments.

The inhibitory effects of the* S. flavescens* extract and individual prenylated flavonoids on eight CYP isoforms were determined by measuring the IC_50_ values. CYP isoform-selective substrates were used for this experiment as described previously [21]. Inhibition of CYP activity was determined by evaluating the net signal change between naïve CYP reactions and test reactions.* S. flavescens *extract displayed strong inhibition of CYP2C8, moderate inhibition of CYP2C9 and CYP2C19, and weak inhibition of CYP2B6, CYP3A4, and CYP2C8 (Table 2 and Figure 3). CYP2C8 was most strongly inhibited by the extract with IC_50_ of 1.42 *µ*g/mL. In human liver microsomes preincubated with the extract in the presence of an NADPH-generating system for 30 minutes, the inhibition of CYP2B6 and CYP3A4 was increased 10- to 50-fold, with IC_50_ values of 0.7 *µ*g/mL and 6.2 *µ*g/mL, respectively. These findings suggest that some active component within the extract inhibits CYP2B6 and CYP3A4 via mechanism-based inactivation. The IC_50_ values of the extract and prenylated flavonoids kushenol A, kushenol C, kushenol I, kushenol M, leachianone A, and sophoraflavone G are listed in Table 2. CYP3A4 was not inhibited by the prenylated flavonoids, even at 50 *µ*M, when microsomes were not preincubated with the flavonoids. However, CYP3A4 was strongly inhibited by kushenol C, kushenol I, kushenol M, leachianone A, and sophoraflavone G in microsomes preincubated with the flavonoids, with IC_50_ values < 5 *µ*M. CYP2B6 activity was more strongly inhibited by kushenol I, leachianone A, and sophoraflavone G after preincubation with the flavonoids, displaying IC_50_ values < 0.5 *µ*M. CYP2A6 and CYP2D6 were not inhibited by the flavonoids regardless of preincubation. CYP2C8 was inhibited by all test flavonoids after preincubation, although kushenol I, leachianone A, and sophoraflavone G showed similar inhibitory effects without preincubation. Kushenol I and sophoraflavone G also inhibited CYP2C9 regardless of preincubation, and kushenol A and leachianone A inactivated CYP2C9. Similar to the inhibition profiles of CYP2B6 and CYP3A4 in microsomes, kushenol I also more strongly inhibited bupropion 6-hydroxylation and midazolam 1'-hydroxylation activities in recombinant CYP2B6 and CYP3A4, respectively, when CYPs were preincubated with kushenol I in the presence of the NADPH-generating system (Figure 4).

### 3.3. Inactivation of CYP3A4 by* S. flavescens* Extract and Kushenol I

**Figure 5:**
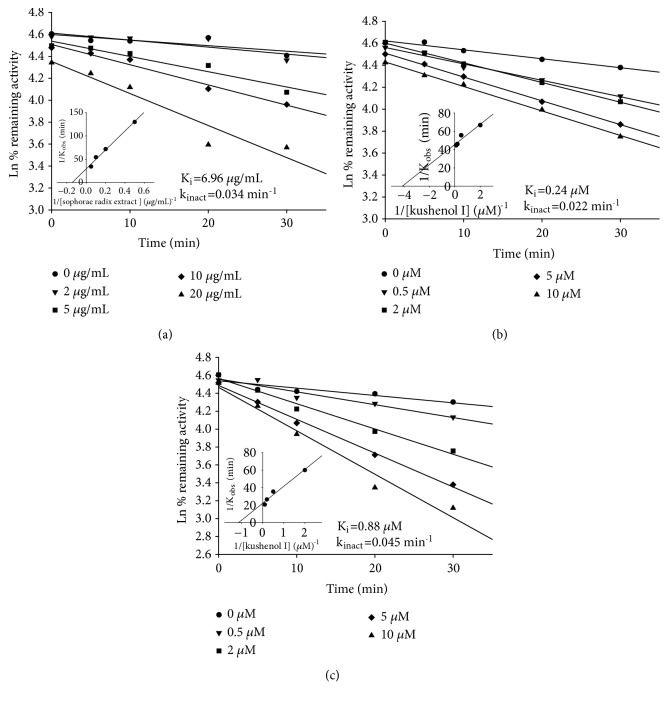
Concentration- and time-dependent inactivation of human liver microsomal CYP3A4 by* S. flavescens* extract (a) and kushenol I (b) and recombinant CYP3A4 by kushenol I (c) in the presence of an NADPH-generating system. The logarithm of the percentage of remaining activity (related to time 0 in the presence of solvent alone) was plotted as a function of time; the corresponding double reciprocal plots for the inactivation rate and the concentration of* S. flavescens* extract or kushenol I are shown. The* K*_*i*_ and* k*_*inact*_ values were obtained from the reciprocals of the x- and y-intercepts, respectively. Each data point represents the mean of duplicate experiments.

The effects of the concentration and incubation time of* S. flavescens *extract and kushenol I on CYP3A4 inhibition were assessed. We chose kushenol I for these experiments as it was the most abundant prenylated flavonoid in the extract. Both* S. flavescens *extract and kushenol I demonstrated time- and concentration-dependent inhibition of CYP3A4 in human liver microsomes (Figures 5(a) and 5(b)). Preincubation of the test materials in the absence of NADPH abolished the time-dependent inhibitory effect, suggesting mechanism-based inactivation. The* K*_*i *_and* k*_*inact*_ values of* S. flavescens *extract were 6.96 *µ*M and 0.034/min for CYP3A4, respectively, and 0.24 *µ*M and 0.022/min for CYP3A4, respectively. The efficiency of CYP3A4 inactivation was assessed by the ratio of* k*_*inact *_to* K*_*i*_, which was 0.0049 for* S. flavescens *extract and 0.092 for kushenol I. These findings suggest that kushenol I inhibits CYP3A4 more efficiently than does the extract. Kushenol I also showed time- and concentration-dependent inhibition of recombinant CYP3A4 with the* K*_*i *_and* k*_*inact*_ values of 0.88 *µ*M and 0.045/min, respectively (Figure 5(c)).

## 4. Discussion

We evaluated the effects of* S. flavescens *extract and its prenylated flavonoids (kushenol A, kushenol C, kushenol I, kushenol M, leachianone A, and sophoraflavone G) on the activities of different CYP isoforms in human liver microsomes using CYP isoform-selective substrates. Considering the central role of CYP enzymes in drug metabolism and clearance, knowledge of the effects of herbal extracts and their active ingredients on CYP enzymes is critical to understanding herb–drug interactions in a clinical setting.* S. flavescens *was traditionally used as a decoction or powder, obtained from its dried roots (Kushen). More than 200 compounds have been identified in* S. flavescens*, with alkaloids and flavonoids exerting the observed pharmacological effects of this medicine [2, 24]. Among the flavonoids, prenylated flavonoids such as kushenols, leachianones, and sophoraflavones were found to exert several pharmacological properties [2, 5, 7]. All of the prenylated flavonoids evaluated in this study were identified in* S. flavescens *extract, although flavonoids may be preferentially extracted by 70% ethanol compared with alkaloids.

Our results indicated that the ethanolic extract of* S. flavescens *inhibited CYP2C8 most strongly, followed by CYP2C9, CYP2C19, CYP3A4, and CYP2B6, but did not inhibit CYP1A2, CYP2A6, or CYP2D6, in human liver microsomes. However, the IC_50_ values of the extract for inhibition of CYP2B6 and CYP3A4 were significantly decreased 10- to 50-fold in microsomes preincubated with the extract in the presence of NADPH, suggesting that the extracts inhibited CYP2B6 and CYP3A4 via mechanism-based inactivation (Table 2, Figure 4). Mechanism-based inactivation of CYP3A4 was further confirmed by kinetic analysis, which revealed* K*_*i *_and* k*_*inact*_ values of 6.96 *µ*g/mL and 0.034/min, respectively. The prenylated flavonoids evaluated in this study exhibited different inhibitory potential toward different CYP isoforms. Kushenol I, which was the most abundant of the prenylated flavonoids in the extract, inhibited CYP2C8 and CYP2C9 reversibly but inhibited CYP2B6 and CYP3A by mechanism-based inactivation. On the other hand, sophoraflavone G reversibly inhibited CYP1A2, CYP2B6, CYP2C8, and CYP2C9, whereas only CYP3A4 was irreversibly inhibited by this flavonoid. Inactivation of CYP3A by kushenol I was also observed, with* K*_*i*_ and* k*_*inact*_ values of 0.242 *µ*M and 0.022/min, respectively. Iwata et al. [25] reported that the methanolic fraction of* Sophora* radix does not inhibit CYP2D6 or CYP3A4 in human liver microsomes. These results are consistent with our findings of limited CYP2D6 and CYP3A4 inhibition in microsomes without preincubation with the extract. Previous research showed that sophocarpine from* S. flavescens *inhibited CYP3A4 in a time-dependent manner and competitively inhibited CYP2C9 [16]. Prenylated flavonoids from* Humulus lupulus *almost completely inhibited CYP1A1 and CYP1B1 activities at a concentration of 10 *µ*M [26]. The prenylated flavone isoxanthohumol also showed time-dependent inactivation of CYP1A2 [17]. Yilmazer and his colleagues [26] demonstrated that xanthohumol is transformed to a diol metabolite, presumably via an epoxide intermediate. The epoxide intermediate generated during the enzymatic reaction may be responsible for inactivation of the CYP enzymes.

The different effects of the active components in* S. flavescens *on CYP isoforms may complicate findings in experimental rats. Oral administration of* S. tonkinensis* extract to rats for 14 days resulted in increased plasma levels of bupropion and omeprazole, which were metabolized mainly by CYP2B and CYP2C, whereas the extract did not significantly modulate CYP1A or CYP3A activity [27]. In addition, administration of* S. flavescens *extract to rats for 7 days increased the plasma levels of phenacetin, omeprazole, and tolbutamide, suggesting inhibition of CYP1A and CYP2C activities [15]. Contrarily, concomitant oral administration of* S. flavescens *extract to rats for 7 days significantly decreased the concentration of plasma indinavir, which is primarily metabolized by CYP3A, whereas the ethyl acetate fraction of* S. flavescens* had no effect [20]. Dose-dependent increases in CYP1A2, CYP2B, and CYP3A activities were reported in rats treated with* S. flavescens *extract, as demonstrated by CYP isoform-selective activity and western blot analysis [18]. CYP3A4 mRNA expression was induced by* S. flavescens *aqueous extract in HepaRG and DPX2 cells via activation of the pregnane X receptor and the compounds N-methylcytisine partially contributed to CYP3A4 induction [28]. Yuan and his colleagues reported that matrine and oxymatrine induce CYP2B, but not CYP3A in experimental rats [17]. Because CYP inhibitors and inducers exist in* S. flavescens, *modulation of CYP isoforms by* S. flavescens* extract may be dependent on the preparation methods of the extract as well as experimental design.

Although the* S. flavescens *extract used in this experiment was prepared with 70% ethanol, and flavonoids may be preferentially extracted compared with alkaloids, the extract inhibited CYP2B6, CYP2C8, CYP2C9, CYP2C19, and CYP3A4 in human liver microsomes. In particular, CYP2B6 and CYP3A4 were inhibited by a mechanism-based mode of inhibition, suggesting that these CYPs may be significantly inhibited by administration of the extract to humans. The mechanism-based inactivation of CYP3A4 was confirmed using the prenylated flavonoid kushenol I. Given the inhibitory effects of prenylated flavonoids along with inductive effects of alkaloids, the overall modulation of CYP activity by* S. flavescens *extract in humans may be complex and dependent on treatment formulation and duration. To the best of our knowledge, no clinical trials have been conducted to evaluate the possible interactions between* S. flavescens* extract and concomitantly administered drugs. Therefore, clinical trials to investigate these herb–drug interactions are required to better predict the effects of* S. flavescens* extract on concomitantly administered drugs in humans.

## 5. Conclusion

In summary,* S. flavescens *extract strongly and reversibly inhibited CYP2C8, CYP2C9, and CYP2C19. CYP2B6 and CYP3A4 were inhibited by the extract via mechanism-based inactivation. The constituents kushenol A, kushenol C, kushenol M, kushenol I, leachianone A, and sophoraflavone G also displayed CYP inhibition, which was dependent on the selectivity of these compounds for the CYP isoforms and on the mode of inhibition. Considering the inhibitory effects of* S. flavescens *extract and its prenylated flavonoids on CYP enzymes, clinical interaction with coadministered drugs that are metabolized by CYP enzymes, especially CYP2B6 and CYP3A4, cannot be excluded. The potential interactions between* S. flavescens* extract and common drugs need further evaluation in human clinical trials.

## Figures and Tables

**Table 1:** . CYP isoform-selective substrates, concentrations, and corresponding metabolites.

CYP isoform	Substrate	Concentration used (*μ*M)	Metabolites
1A2	Phenacetin	50	Acetaminophen
2A6	Coumarin	5	7-Hydroxycoumarin
2B6	Bupropion	50	6-Hydroxybupropion
2C8	Rosiglitazone	5	Hydroxyrosiglitazone
2C9	Diclofenac	10	Hydroxydiclofenac
2C19	*S*-Mephenytoin	80	4-Hydroxymephenytoin
2D6	Dextromethorphan	5	Dextrorphan
3A4	Midazolam	2	1-Hydroxymidazolam
			-

**Table 2:** IC_50_ values of the standardized hop extract and individual prenylated flavonoids for inhibition of specific CYP isoforms in human liver microsomes.

CYP isoform	Preincubation	IC_50_ value						
		*S. flavescens* extract (*μ*g/mL)	Kushenol A (*μ*M)	Kushenol C (*μ*M)	Kushenol I (*μ*M)	Kushenol M (*μ*M)	Leachianone A (*μ*M)	Sophoraflavone G (*μ*M)
1A2	-	>100	>50	5.00	>50	>50	>50	2.26
	+	54.6	>50	4.86	>50	>50	>50	0.17
2A6	-	>100	>50	>50	>50	>50	>50	>50
	+	>100	>50	>50	>50	>50	>50	>50
2B6	-	66.5	>50	>50	>50	>50	5.74	2.42
	+	0.7	>50	>50	0.12	>50	0.28	0.07
2C8	-	1.42	>50	>50	0.67	>50	0.75	0.32
	+	0.49	0.11	7.07	0.14	0.28	0.33	0.09
2C9	-	13.6	>50	9.3	7.35	>50	>50	1.25
	+	12.3	2.5	6.9	5.03	>50	2.05	0.42
2C19	-	19.1	>50	>50	>50	>50	>50	N.D
	+	18.7	>50	6.6	>50	>50	3.13	N.D
2D6	-	>100	>50	>50	>50	>50	>50	>50
	+	73.4	>50	>50	>50	>50	>50	>50
3A4	-	51.0	>50	>50	>50	>50	>50	>50
	+	6.2	>50	3.95	0.57	1.29	0.69	0.78

## Data Availability

The data used to support the findings of this study are available from the corresponding author upon request.
